# Microtubule Organizing Centers Contain Testis-Specific γ-TuRC Proteins in Spermatids of Drosophila

**DOI:** 10.3389/fcell.2021.727264

**Published:** 2021-09-29

**Authors:** Elham Alzyoud, Viktor Vedelek, Zsuzsánna Réthi-Nagy, Zoltán Lipinszki, Rita Sinka

**Affiliations:** ^1^Department of Genetics, University of Szeged, Szeged, Hungary; ^2^Faculty of Science and Informatics, Doctoral School of Biology, University of Szeged, Szeged, Hungary; ^3^Biological Research Centre, Institute of Biochemistry, MTA SZBK Lendület Laboratory of Cell Cycle Regulation, Eötvös Loránd Research Network (ELKH), Szeged, Hungary

**Keywords:** Drosophila, spermatogenesis, γ-TuRC, microtubules, MTOC, basal body, mitochondria

## Abstract

Microtubule nucleation in eukaryotes is primarily promoted by γ-tubulin and the evolutionary conserved protein complex, γ-Tubulin Ring Complex (γ-TuRC). γ-TuRC is part of the centrosome and basal body, which are the best-known microtubule-organizing centers. Centrosomes undergo intensive and dynamic changes during spermatogenesis, as they turn into basal bodies, a prerequisite for axoneme formation during spermatogenesis. Here we describe the existence of a novel, tissue-specific γ-TuRC in Drosophila. We characterize three genes encoding testis-specific components of γ-TuRC (t-γ-TuRC) and find that presence of t-γ-TuRC is essential to male fertility. We show the diverse subcellular distribution of the t-γ-TuRC proteins during post-meiotic development, at first at the centriole adjunct and then also on the anterior tip of the nucleus, and finally, they appear in the tail region, close to the mitochondria. We also prove the physical interactions between the t-γ-TuRC members, γ-tubulin and Mozart1. Our results further indicate heterogeneity in γ-TuRC composition during spermatogenesis and suggest that the different post-meiotic microtubule organizing centers are orchestrated by testis-specific gene products, including t-γ-TuRC.

## Introduction

Microtubule-organizing centers and microtubule cytoskeleton contribute to the process of cellular differentiation, cell division and the organization of flagella and cilia. The centrosome is the best-understood and main microtubule-organizing center (MTOC) of eukaryotic dividing cells, and is composed of two centrioles surrounded by pericentriolar material (Wiese and Zheng, 1999; Lattao et al., 2017). Apart from the centrosome, other non-centrosomal MTOCs (ncMTOCs) exist to fulfil specific functions on the mitochondria, Golgi, nuclear envelope, or cell cortex. Both the centrosome and the ncMTOCs can recruit the multiprotein γ-TuRC to the nucleation site. γ-tubulin exists in two evolutionary conserved complexes: the γ-Tubulin Small Complex (γ-TuSC) and γ-TuRC (Wiese and Zheng, 1999; Tillery et al., 2018). Despite the importance of γ-TuRC in γ-Tubulin binding and microtubule nucleation, the core γ-TuRC proteins are represented by only one ortholog in different species so far. The stabilization of the core γ-TuRC was suggested by binding with additional γ-TuRC interacting proteins, such as Mozart1 (Mzt1) and Mozart2, Nedd1 or the γ-TuRC tethering CDK5RAP2 (Liu et al., 2021). In Drosophila γ-TuSC is composed of two γ-tubulins and two Grip proteins, Grip84 and Grip91; while γ-TuRC comprises multiple γ-TuSC proteins and three or four additional Grip proteins, Grip75, Grip128, Grip163, and Grip71 (Gunawardane et al., 2000; Veìrollet et al., 2006). It was shown that both the CDK5RAP2 ortholog Cnn and the recently identified Mzt1 can bind to components of the core γ-TuRC in Drosophila (Zhang and Megraw, 2007; Tovey et al., 2018). The testis-specific isoform of Cnn, CnnT was shown to support the formation of MTOCs on the mitochondrial surface, while Mzt1 was shown to concentrate on the centriole adjunct of spermatids (Chen et al., 2017; Tovey et al., 2018).

Gene duplication is a major mechanism in molecular evolution, providing the possibility of neofunctionalization and subfunctionalization of the duplicated genes. Many metabolic enzymes and protein-degradation-related genes have been shown to have duplicated retrogenes with testis-specific transcript enrichment patterns in the late stages of spermatogenesis (Porcelli et al., 2007; Belote and Zhong, 2009; White-Cooper and Bausek, 2010).

Testis-specific gene products contribute to specialized sperm-specific organelle formation in late spermiogenesis, such as the elongated mitochondria, the acrosome or centriole adjunct (Dallai et al., 2016; Vedelek et al., 2016; Chen et al., 2017; Laurinyecz et al., 2019). As the spermatids begin to elongate, the nebenkern unfurls and forms two mitochondrial derivatives, which elongate with the cooperation of cytoplasmic non-axonemal microtubules and run along the sperm tail (Noguchi et al., 2011). Centrosomes transform into basal bodies and the centrioles become exceptionally long during spermatocyte maturation, eventually giving rise to a 1.8 mm long flagellar axonemes after the meiotic divisions (Fabian and Brill, 2012). Several basal body or centriole adjunct components of the spermatids are not present in the mature sperm, suggesting their intensive reduction in late spermiogenesis (e.g., γ-Tubulin, Sas4, Sas6) (Raynaud-Messina et al., 2001; Blachon et al., 2014). Since sperm elongation and nuclear shaping largely depend on the microtubule cytoskeleton, microtubule nucleation must be strictly regulated to guarantee the correct formation of microtubule networks in the different stages of sperm development. Interestingly, many Drosophila mutants for centrosome, basal body or centriole adjunct components are viable, but male sterile, suggesting that male germline development is very sensitive to alterations in these organelles, making it an ideal system to study the components of MTOCs through cell differentiation.

In this study, we describe the presence and dynamic post-meiotic protein localization of alternative γ-TuRCs during spermatogenesis in *Drosophila melanogaster*. We identified three testis-specific γ-TuRC (t-γ-TuRC) proteins, t-Grip84, t-Grip91, and t-Grip128 and we show that from the round spermatid stage onward, the three t-γ-TuRC proteins start to localize at the centriole adjunct, then also to the nuclear tip, and finally surrounding the mitochondria of the elongating cyst. We demonstrate that the identified complexes colocalize and bind directly to both γ-tubulin and Mzt1, suggesting a transition of these proteins between the somatic and testis-specific complex. Our work proves the existence of different types of γ-TuRCs in the spermatogenesis of *Drosophila melanogaster*.

## Results

### Identification and Phenotypic Characterization of t-γ-TuRC Members

**FIGURE 1:**
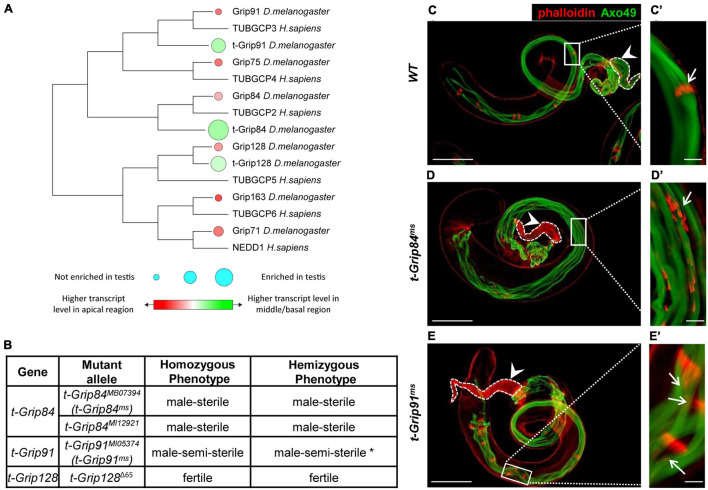
Identification and phenotypic characterization of t-γ-TuRC members. **(A)** The phylogenetic tree shows conservation among Drosophila and human γ-TuRC proteins. Transcript accumulation in the apical region (red) and basal region (green) and testis enrichment of γ-TuRC members are labelled. **(B)** Phenotypic characterization of t-γ-TuRC mutants. *70% of *t-Grip91^*ms*^* males are sterile. (See also Supplementary Figures 2B,C). **(C–E)** Polyglycylated elongated axonemes are visualized by AXO49 staining (green) and individualization complexes stained with Phalloidin (red). Axoneme elongation of *t-Grip84^*ms*^* and *t-Grip91^*ms*^* mutants is similar to WT. **(C’)** Movement of the investment cones are synchronized (arrow) and **(C)** seminal vesicle is filled with mature sperm (arrowhead) in WT testis. **(D’,E’)** Scattered investment cones (arrows) and **(D,E)** empty seminal vesicles (arrowhead) are present in *t-Grip84^*ms*^* and *t-Grip91^*ms*^* mutants. Scale bars: C-E 100 μm, **(C’–E’)** 20 μm.

We previously conducted a transcriptomic analysis of the different regions of Drosophila testis and identified members of several protein families (including γ-tubulin-containing complexes) with high transcript accumulation in the basal end of the testis (Vedelek et al., 2018). Transcripts of the somatic γ-TuRC genes (Grip84, Grip91, Grip75, Grip128, Grip163, and Grip71) are present mainly in the apical end of the testis, based on RNA sequencing data (Figure 1A; Vedelek et al., 2018). Interestingly, we identified three testis-specific Grip paralogs (CG7716 (t-Grip84), CG18109 (t-Grip91), and CG32232 (t-Grip128)) with high transcript accumulation in the basal end of the testis, suggesting that they are components of an uncharacterized testis-specific γ-TuRC (Figure 1A). We compared the somatic and testis-specific γ-TuRC proteins by protein sequence alignment and also we constructed a phylogenetic tree to visualize the relation between the γ-TuRC proteins (Figure 1A; Supplementary Figure 1).

To investigate the function of the t-γ-TuRC coding genes, we analyzed their mutant phenotype. We collected the available mutants of *t-Grip84 (t-Grip84^*MB07394*^* (thereafter *t-Grip84^*ms*^*), *t-Grip84^*MI12921*^), t-Grip91 (t-Grip91^*MI05374*^* (thereafter *t-Grip91^*ms*^*)) and generated a null mutant allele for *t-Grip128* using CRISPR-Cas9 gene editing (*t-Grip128^Δ65^*). By measuring the relative gene expression in homozygous *t-Grip84^*ms*^*, *t-Grip91^*ms*^* and *t-Grip128^Δ65^* testis, we found a dramatic reduction of the gene products in all three mutants (Supplementary Figure 2A). Despite containing a deletion of 3359 bp, which removes the start codon and most of the coding region (1–891aa) of *t-Grip128*, homozygous *t-Grip128^Δ65^* males are fertile, suggesting that *t-Grip128* is not essential for normal male fertility (Figure 1B and Supplementary Figure 2B). We tested the age-dependent fertility of *t-Grip128^Δ65^* mutants and found a comparable number of offspring in the control, suggesting a redundant role in male fertility for *t-Grip128* (Supplementary Figure 2C). However, both *t-Grip84^*ms*^* and *t-Grip84^*MI12921*^* are male-sterile, while *t-Grip91^*ms*^* males are semi-sterile in homozygotes and hemizygotes (Figure 1B and Supplementary Figure 2B). We found empty seminal vesicles, lacking mature sperm in *t-Grip84^*ms*^*, whereas a reduced number of sperms is present in *t-Grip91^*ms*^* seminal vesicles (Figures 1C–E).

During cyst elongation, long axonemes form parallel with the elongated mitochondria, in close connection with the elongated nucleus, followed by cyst individualization which is orchestrated by actin-containing individualization complexes (IC). To understand the function of t-γ-TuRC proteins during spermatogenesis, we investigated the morphology of the *t-Grip84^*ms*^* and *t-Grip91^*ms*^* mutant testes. We analyzed the different developmental stages of spermatogenesis and found that the mitotic and meiotic divisions were normal in both t-γ-TuRC mutants, resulting in wild type round spermatids with equal-sized nebenkern and nucleus, in a 1:1 ratio (Supplementary Figures 2D–G). First, we tested the presence and distribution of polyglycylated tubulin, a hallmark of the fully elongated, developed axoneme of the spermatid at the onset of individualization (Bre et al., 1996). We found that, similar to the wild type, the axonemes of both mutants contain polyglycylated tubulin, suggesting normal cyst elongation and axoneme formation (Figures 1C–E). Next, we tested the individualization of the spermatids (Figures 1C–E). Visualization of the actin cones of the IC revealed that, although the formation of the actin cones is not disturbed, they become severely dispersed in the sterile *t-Grip84^*ms*^*. We observed a weaker phenotype in *t-Grip91^*ms*^* mutant cysts, but abnormally distributed actin cones were still present (Figures 1C’–E’ and Supplementary Figure 2H). These results strongly suggest that t-γ-TuRC is not essential during the early stages of spermatogenesis or for the formation of the axoneme, and the asynchronous movement of the ICs of the elongated spermatids is probably a secondary consequence of the disturbed spermiogenesis.

### Localization of t-γ-TuRC Members During Spermatogenesis

**FIGURE 2:**
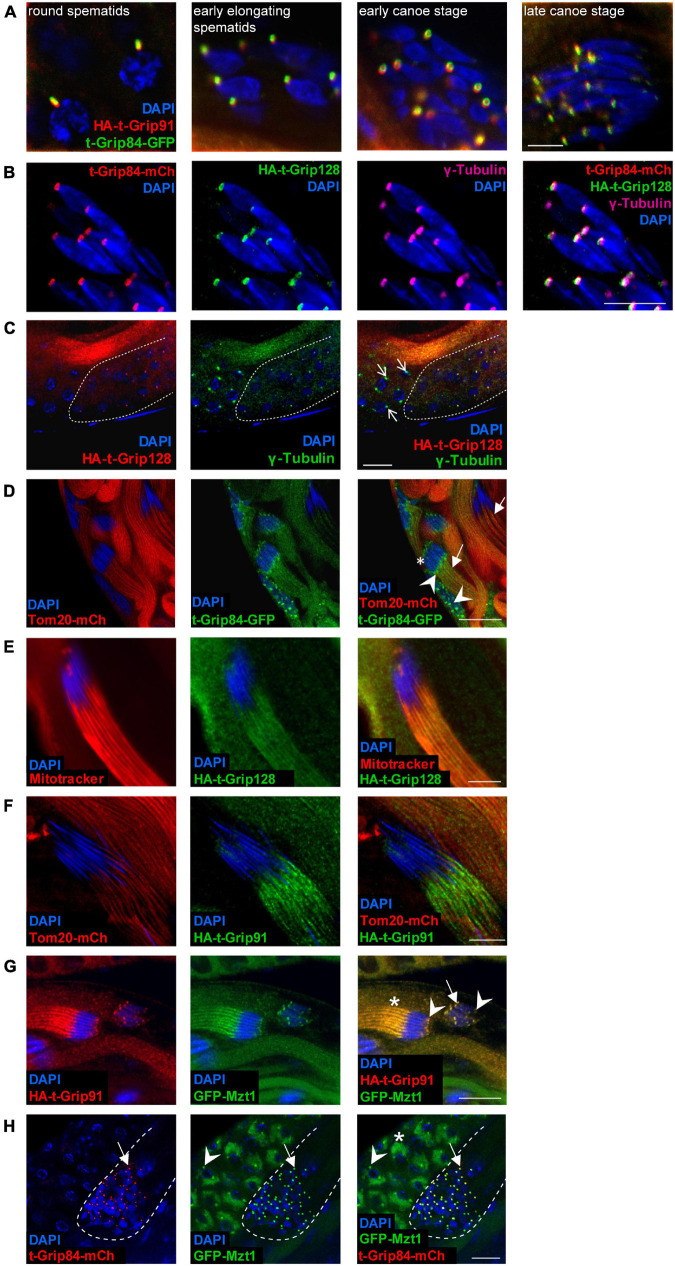
Localization of t-γ-TuRC proteins during spermatogenesis. **(A)** HA-t-Grip91 (red) colocalizing with t-Grip84-GFP (green) in round spermatids, early elongating, early canoe and late canoe stage spermatids. **(B)** t-Grip84-mCh (red), colocalizing with HA-t-Grip128 (green), and also with γ-tubulin (magenta) at the centriole adjunct in canoe stage elongating spermatids. Nuclei are visualized by DAPI (blue). **(C)** HA-t-Grip128 (red) is not present on the centrosome of meiotic spermatocytes (arrow), but present and show an overlapping pattern with γ-Tubulin (green) in round spermatids (the cyst highlighted with dashed line). **(D)** In addition to the centriole adjunct localization (arrowhead) t-Grip84-GFP is localizing to the nuclear tip (asterisk) and close proximity to the mitochondria, labelled by Tom20-mCh (arrows) in the elongating cysts. **(E,F)** HA-t-Grip128 and HA-t-Grip91 are localizing close to the mitochondria (stained with Mitotracker (red) and labelled by Tom20-mCh, respectively) of the late elongating spermatids. **(G)** HA-t-Grip91 (red) colocalizing with GFP-Mzt1 (green) on the centriole adjunct (arrow) and at the tip of the nuclei (arrowhead) in the canoe stage and near the mitochondria (asterisk) in the needle stage spermatids. **(H)** t-Grip84-mCh and GFP-Mzt1 are colocalizing on the centriole adjunct (arrow) in the post-meiotic cysts (highlighted by the white dashed lines), while GFP-Mzt1 is present on the centrosome (arrowhead) and close to the mitochondria (star) of meiotic spermatocytes. Scale bars: A 5 μm, **(C,D,G,H)** 20 μm, **(B,E,F)** 10 μm.

**FIGURE 3:**
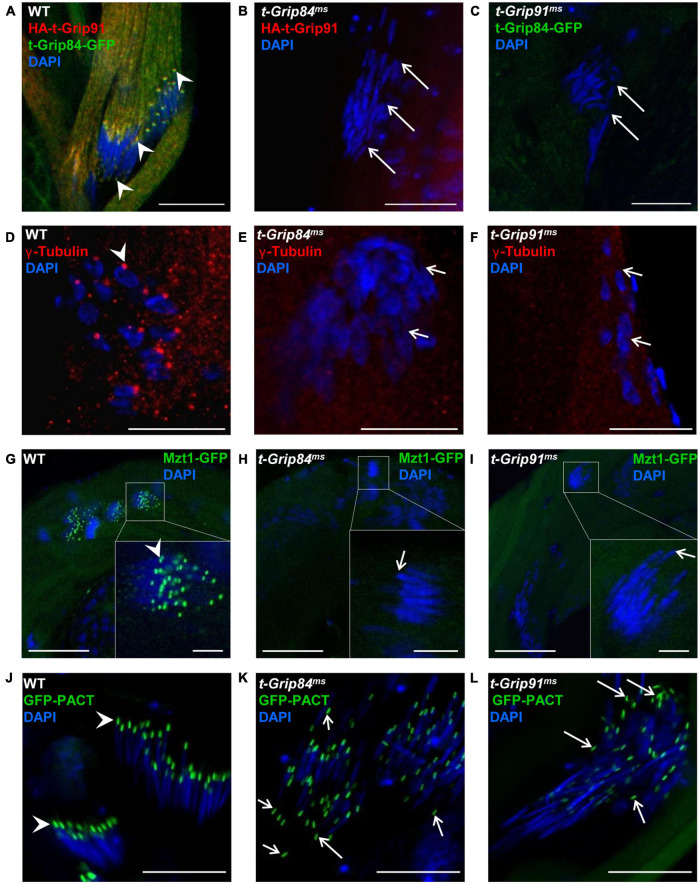
Localization of centriole adjunct components in t-γ-TuRC mutants. **(A–C)** HA-t-Grip91 and t-Grip84-GFP are colocalized on the centriole adjunct and nuclear tip (arrows) in WT spermatids, while HA-t-Grip91 became cytoplasmic in *t-Grip84^*ms*^* and t-Grip84-GFP in *t-Grip91^*ms*^* mutant spermatids. **(D–I)** γ-tubulin (red, arrowhead) and Mzt1-GFP (green, arrowhead) are mislocalized in *t-Grip84^*ms*^* and *t-Grip91^*ms*^* mutants. **(J–L)** GFP-PACT is on the basal body in WT, *t-Grip84^*ms*^* and *t-Grip91^*ms*^* elongating spermatids, but GFP-PACT signal detached from the nucleus in *t-Grip84^*ms*^* and *t-Grip91^*ms*^* spermatids (arrows). Statistical analysis is in Supplementary Figure 5D. Scale bars: **(A–F)** and **(J–L)** 20 μm, G-I 50 μm, insets 10 μm.

To better understand the expression pattern and subcellular distribution of the t-γ-TuRC components, we generated transgenic proteins fused with different protein tags. We established *t-Grip84-mCherry* (*t-Grip84-mCh*), *t-Grip84-GFP, HA-t-Grip91*, and *HA-t-Grip128* transgenic lines, under the control of their own promoters (genomic locus surrounding the 5′ end of the coding region of each gene). We were able to rescue the male sterility with the tagged version of *t-Grip84* or *t-Grip91*, indicating the functionality of the transgenes (Supplementary Figure 2B). The presence of the t-γ-TuRC proteins could only be detected after the meiotic divisions, exclusively in the germline cells of the cysts. t-Grip84-mCh or t-Grip84-GFP, HA-t-Grip91, and HA-t-Grip128 signals appear first in the round spermatids and localize to the centriole adjunct during nuclear elongation (Figures 2A–C,HSupplementary Figures 3A,C). Centriole adjunct localization of the t-γ-TuRC proteins was further confirmed by simultaneous labelling of t-Grip84-mCh/t-Grip84-GFP, HA-t-Grip91, and HA-t-Grip128 with γ-tubulin. We concluded that t-γ-TuRC members colocalize with γ-tubulin and with each other at the centriole adjunct (Figures 2A–C and Supplementary Figure 3A). The centriole adjunct localization of t-γ-TuRC persists in the elongating spermatid stage but diminishes in the fully elongated cysts (Supplementary Figure 3B). We observed additional localization signals of the t-γ-TuRC proteins through the development of the cysts, on the anterior tip of the elongating nuclei and on the tail region, near the mitochondria of the late elongating spermatids (Figures 2D–G and Supplementary Figures 3D,E). We confirmed HA-t-Grip91, HA-t-Grip128 and t-Grip84-GFP potential mitochondrial association by staining with Mitotracker or coexpressing with the mitochondrial outer membrane protein Tom20-mCh (Figures 2D–F) (Zhang et al., 2016). Interestingly, Mzt1, the Grip91- and Grip128-interacting protein is known to exhibit a partly similar localization pattern to the newly identified t-γ-TuRC members (Tovey et al., 2018). To test the colocalization of Mzt1 and the t-γ-TuRC proteins, we engineered an endogenous promoter-driven GFP-Mzt1 transgene. We confirmed the previously published centrosomal and centriole adjunct localization of GFP-Mzt1 (Figure 2H; Supplementary Figure 3F) (Tovey et al., 2018). We also found additional GFP-Mzt1 accumulation in a close association with the mitochondria during the meiotic division of spermatocytes using the mitochondrial marker Tom20-mCh (Figure 2H and Supplementary Figure 4A). We could not detect GFP-Mzt1 signal on the surface of the mitochondria of the onion stage and early elongating spermatids (Supplementary Figures 4B,C; Tovey et al., 2018). We found GFP-Mzt1 colocalization with all three t-γ-TuRC proteins after meiosis, from the round spermatid stage (Figures 2G,H and Supplementary Figure 3D). Similarly to the localization of t-γ-TuRC proteins, the GFP-Mzt1 signal was detected at the centriole adjunct, the tip of the nuclei, and additionally resides closely to the mitochondria of the late elongating spermatids (Figure 2G; Supplementary Figures 3D, 4B–D). This kind of diverse and overlapping distribution of t-γ-TuRC members and Mzt1 in spermatids strongly supports the existence and necessity of multiple MTOCs in post-meiotic stages of Drosophila spermatogenesis.

Next, we tested the mutual dependence of the diverse localization of the t-γ-TuRC proteins. We found that neither HA-t-Grip91 nor t-Grip84-GFP localizes to the centriole adjunct or nuclear tip in the round or early elongating spermatids of *t-Grip84^*ms*^* or *t-Grip91^*ms*^* mutants, respectively (Figures 3A–C; Supplementary Figures 5H,I). Afterward, we analyzed the distribution of γ-tubulin and Mzt1, the two potential interactor proteins of the complex in the mutants. We found that both γ-tubulin and GFP-Mzt1 are present on the centrosome of spermatocytes in *t-Grip84^*ms*^* and *t-Grip91^*ms*^* mutants, however, from the round spermatid stage onward, their localizations disappear and, they become cytoplasmic (Supplementary Figures 3F–N and Figures 3D–I). These results provide additional evidence that t-γ-TuRC function is restricted to the post-meiotic stages and is not essential for centrosomal recruitment of the γ-TuRC interacting proteins. These findings raised the question of whether the lack of *t-Grip84* or *t-Grip91* resulted in a general disintegration of the centriole adjunct and basal body. We tested the localization of three basal body proteins, the centriole/pericentriolar material protein PACT, the centriole component Ana1 and Asterless in *t-Grip84^*ms*^* and *t-Grip91^*ms*^* mutants to assess the impact of the lack of t-γ-TuRC proteins (Martinez-Campos et al., 2004; Fu et al., 2016; Galletta et al., 2020). GFP-PACT, Ana1 and Asterless were found on the basal body of *t-Grip84^*ms*^* and *t-Grip91^*ms*^* mutant spermatids. However, we observed their nuclear scattering during the later stages of individualization, as well as the partial detachment of the GFP-PACT, Ana1 and Asterless labelled basal body from the nucleus in both mutants (Figures 3J–L and Supplementary Figures 5A–K).

The t-γ-TuRC members influence each other’s localization and despite normal axoneme assembly, basal body attachment to the nucleus and integrity of the cysts is disturbed in the *t-Grip84^*ms*^* and *t-Grip91^*ms*^* mutants. Taken together, these results suggest that the observed abnormalities in the mutants are not due to the disappearance of centriole adjunct or general failure of sperm development.

### Composition of the t-γ-TuRC

**FIGURE 4:**
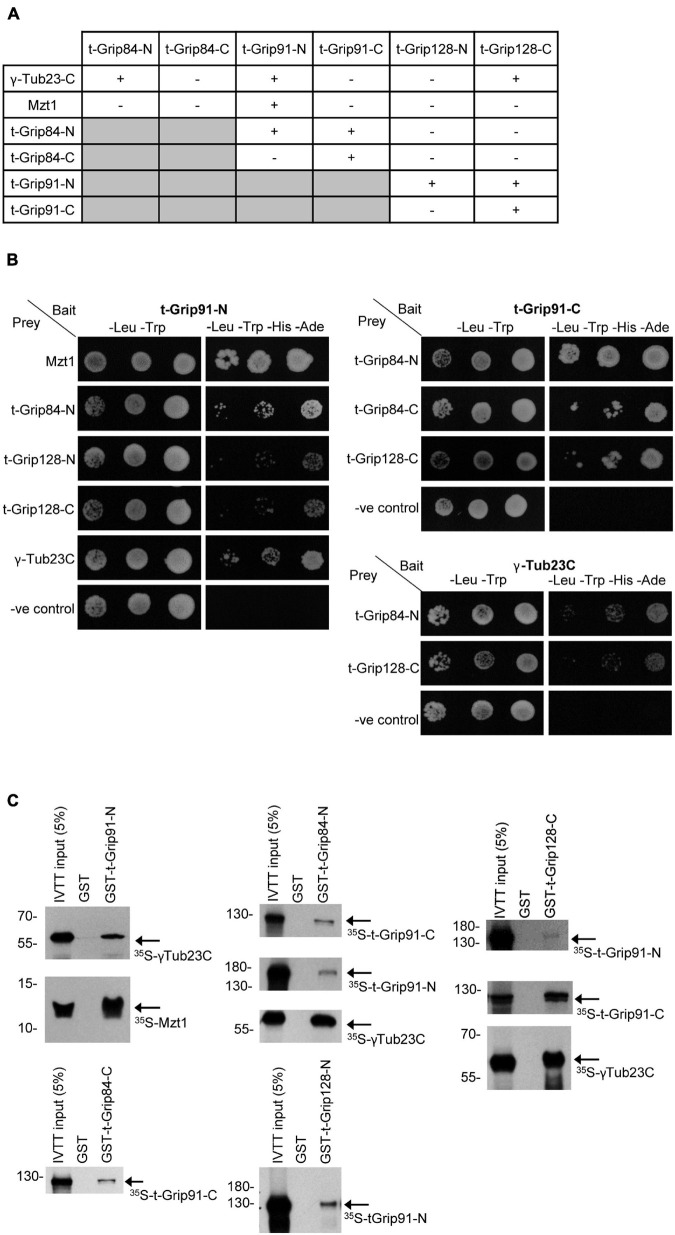
Interaction between the t-γ-TuRC and partner proteins. **(A)** Summary of Y2H analysis between N-and C-terminal t-γ-TuRC proteins with each other and with γ-Tub23-C and Mzt1. **(B)** Mated yeast were plated as 10-fold serial dilutions (left to right) on -Leu, -Trp medium that selective for the bait and prey plasmids and -Leu, -Trp, -His, -Ade medium that selective for the bait, prey, and the interaction between the tested proteins. Negative control (-ve control) is the empty prey with the corresponding bait vector. **(C)** Autoradiography showing the interactions between purified GST-t-γ-TuRC protein fragments and ^35^S-methionine-labelled interacting proteins produced in IVTT reaction.

Together, the above observations suggested that an alternative γ-TuRC could assemble in the post-meiotic stages of spermatogenesis, but its molecular composition remained partly unclear. To address this issue, we first performed a yeast two-hybrid (Y2H) analysis to test the interactions between t-γ-TuRC proteins in every combination and with full-length γ-tubulin and Mzt1 (Figure 4A). Due to the large size of the proteins, we divided all three t-γ-TuRC proteins into an N-terminal and a C-terminal part (Supplementary Figure 6A). We found that t-Grip84-N, t-Grip91-N, and t-Grip128-C bind to the ubiquitously expressed γ-Tub23C. We showed that Mzt1 binds exclusively to the N-terminal of t-Grip91 (Figures 4A,B). Interaction of the N-terminal of both somatic Grip91 and Grip128 with Mzt1 was previously reported, however, the precise location of this interaction during spermatogenesis was not investigated (Tovey et al., 2018). Our results suggest that N-terminus of t-Grip91 could be responsible for the t-γ-TuRC interaction of Mzt1 in the post-meiotic stages. We found that t-Grip84-N interacts with both t-Grip91-N and t-Grip91-C. Moreover, t-Grip91-C shows binding with t-Grip84-C and t-Grip128-C. t-Grip91-N also shows binding with both t-Grip128-N and t-Grip128-C in Y2H assays (Figures 4A,B). To confirm the positive Y2H results, we performed direct binding experiments, in which we individually expressed, purified, and immobilized the GST-tagged version of the N- or C-terminal parts of the t-γ-TuRC proteins (bait). Bait proteins were mixed with ^35^S-methionine-labelled candidates, produced with a coupled *in vitro* transcription-translation (IVTT) system followed by autoradiography to detect physical interactions. Results of the *in vitro* binding experiments supported the Y2H findings (Figure 4C and Supplementary Figure 6). Consequently, we proved with two independent experimental systems that all three t-γ-TuRC proteins could bind with γ-tubulin, but only the N-terminal t-Grip91 shows interaction with Mzt1. Furthermore, the diverse binding of the core t-γ-TuRC proteins with each other implies the possibility of a variable t-γ-TuRC composition, which could contribute to the different localization of the complexes in the late stages of spermatogenesis.

## Discussion

It is understood, that the multiprotein γ-TuRC is a template for microtubule nucleation in various parts of the cell (Tovey and Conduit, 2018). In this study, we have shown that *Drosophila melanogaster* encodes three t-γ-TuRC proteins, t-Grip84, t-Grip91, and t-Grip128 and we identified and characterized the classical mutants of *t-Grip84* and *t-Grip91* and *t-Grip128*. We found that *t-Grip84^*ms*^* and *t-Grip91^*ms*^* are male sterile, while *t-Grip128^Δ65^* males are fertile. It is also known, that *Grip84*, *Grip91* and *γ-tub23C* mutants are lethal, while *Grip75* and *Grip128* mutants are female and male sterile, suggesting that γ-TuSC is essential at each developmental stage and γ-TuRC components have a modulator function in somatic cells (Sunkel et al., 1995; Barbosa, 2000; Bourbon et al., 2002; Vogt, 2006). The male sterility of *Grip75* and *Grip128* mutants manifest in the late post-meiotic stages of spermatogenesis, similar to *t-Grip84^*ms*^* and *t-Grip91^*ms*^* mutants, indicating that the late stages of spermatogenesis are particularly sensitive to alterations in γ-TuRC composition, likely due to the lack of transcriptional compensation mechanisms following meiosis.

The scattered and detached individualization complexes and centrioles in the *t-Grip84^*ms*^* and *t-Grip91^*ms*^* mutants suggest that t-γ-TuRC contribute to the establishment or maintenance of the stable connection between the nucleus and cilia.

The necessity of *Grip128* could explain the normal fertility of *t-Grip128^Δ65^* males, where the product of *Grip128* is probably sufficient for normal sperm development and fertility.

t-γ-TuRC members show a dynamic post-meiotic protein localization pattern in the developing spermatids. We found that from the round spermatid stage onward the three t-γ-TuRC proteins begin to localize at the centriole adjunct, then also to the nuclear tip, and then finally to the surface of the mitochondria of the late elongating cyst, where they colocalize with γ-tubulin and Mzt1 (Chen et al., 2017; Tovey et al., 2018). It was recently shown that γ-Tubulin localizes both to the nuclear tip and the centriole adjunct of the elongating spermatids and could contribute to the nuclear elongation (Riparbelli et al., 2020). The centriole adjunct and apical tip localization of t-γ-TuRC proteins and γ-Tubulin itself indicate that they could contribute to the organization of the perinuclear microtubule network, and to stabilizing the attachment of the axoneme to the nucleus. One may imagine that an MTOC at the nuclear tip could promote nuclear elongation, as well as the organization of the acroblast, the precursor of the acrosome.

This diverse t-γ-TuRC composition implies the possibility of multiple t-γ-TuRCs with diverse localization abilities during the post-meiotic stages of spermatogenesis. It is tempting to speculate that, despite both Mzt1 and γ-tubulin binding to the somatic γ-TuRC prior to the meiotic stages, following meiosis, Mzt1, and probably γ-tubulin, can also be recruited to different MTOCs by the t-γ-TuRC (Supplementary Figure 7).

Two lines of evidence support the hypothesis that the testis-specific splice variant, centrosomin (CnnT), orchestrates the formation of the mitochondrial MTOC by recruitment of γ-TuRCs to the elongating mitochondria. Firstly, CnnT is able to recruit the γ-TuRC proteins GCP2 and GCP5 to the mitochondria in HEK293 cells. Secondly, CnnT itself localizes on the surface of spermatid mitochondria, converting mitochondria into MTOCs during spermatid development (Chen et al., 2017). Taking these results together with earlier findings and the phenotype of the mutants, we suggest that t-Grip128, Mzt1 and CnnT could have moderator or redundant roles, in contrast to t-Grip84, t-Grip91, Grip75, and Grip128, which likely have more fundamental roles in spermatid development (Vogt, 2006; Chen et al., 2017; Tovey et al., 2018). Additional experiments are necessary to test whether CnnT is a direct binding partner of t-γ-TuRC in the post-meiotic stages and to test the significance of the mitochondrial association of t-γ-TuRC and its interactor proteins in the late elongating spermatids.

Targeting γ-tubulin to the elongating spermatids is not dependent on Grip75 or Grip128, despite the γ-tubulin binding capacity of both proteins (Vogt, 2006). While all tested γ-TuRC and t-γ-TuRC proteins could bind to γ-tubulin *in vitro*, the localization of γ-tubulin is disturbed only in the post-meiotic cysts of the *t-Grip84^*ms*^* and *t-*Grip91*^*ms*^* mutants. This strongly suggests that t-Grip84 and t-Grip91 are fundamental in the centriole adjunct, nuclear tip and mitochondrial recruitment of γ-tubulin in the post-meiotic stages. Based on the late spermatogenesis phenotype of *Grip75* and *Grip128* mutants, and the fact that Grip163 (GCP6) was shown to be part of the Drosophila sperm proteome, we can imagine that some of the somatic γ-TuRC proteins could be present in the t-γ-TuRC and inherited through the sperm following fertilization (Vogt, 2006; Wasbrough et al., 2010). We believe that our results open up a new avenue to understanding the composition of alternative γ-TuRCs in different specialized cell types, such as the spermatids, and the function of MTOCs’ heterogeneity.

## Materials and Methods

### Fly Stocks, Mutants the Sperm Following, and Fertility Test

Flies were maintained on cornmeal agar medium at 25°C in standard lab conditions. Fly stocks used in this study were obtained from Bloomington Drosophila Stock Center, unless stated otherwise. GFP-PACT line was obtained from Jordan Raff’s and Tom20-mCherry from Hong Xu’s laboratory (Martinez-Campos et al., 2004; Zhang et al., 2016). *w*^1118^ used as wild type control (3605, BDSC). Mi{MIC}CG7716^*MI12921*^ (58006, BDSC), Mi{ET1}CG7716^*MB07394*^ (26394, BDSC), Df(3L)BSC375, (24399, BDSC), Mi{MIC}CG18109^*MI05374*^ (42318, BDSC), and Df(2L)osp29 (3078, BDSC) mutant stocks were used in this study. The *pCFD4-Grip128-gRNA* line was crossed with the Cas9 source line, y1 M{nos-Cas9.P}ZH-2Aw- (BDSC 54591) to generate deletions in the *t-Grip128* gene. The deletions were identified by PCR and sequencing, and *t-Grip128^Δ65^* line was established. Transgenic constructs for t-Grip84-GFP, t-Grip84-mCh, HA-t-Grip91, HA-t-Grip128 and GFP-Mzt1 were injected into P{CaryP}attP40 (BDSC 25709) or P{CaryP}attP2 (BDSC 8622) fly lines. For fertility tests, individual males were crossed with three *w*^1118^ virgin females. 14 days after crossing, the hatched progeny was counted in every tube. Experiments were on four occasions with > 10 males per condition.

### DNA Constructs

To make *t-Grip128^Δ65^* null mutant alleles, two sgRNAs (t-Grip128_CRISPR1 AAAACAAGTCTTAAACTCA, t-Grip128_ CRISPR2 GATTCCGTCTAAGTTGAGT) were cloned into pCFD4 vector (Addgene, 49411) at Bbs1 site to create a deletion of 3,359 bp and stable transgenic lines were established as described in Port and Bullock (2016) and Port et al. (2014). t-Grip84-mCh, t-Grip84-GFP, HA-t-Grip91, HA-t-Grip128, and GFP-Mzt1 constructs were made by amplifying the 5′ upstream regions (2,134 bp of *t-Grip84*, 970 bp of *t-Grip91*, 2,213 bp of *t-Grip128* and 1,581 bp of *Mzt1*) from genomic DNA, the coding sequence of t-γ-TuRC proteins and Mzt1 from *w*^1118^ testis cDNA (NEB, M0530S).

The purified PCR products (K0502, Thermo Fisher) were cloned into pUASTattB-mCherry, pUASTattB-GFP or pUASTattB-3xHA vectors using HiFi DNA Assembly Master Mix (E2621S, NEB) (Gibson et al., 2009). For YTH analysis t-Grip84s (1–399 aa N-term, 400–728 aa C-term), t-Grip128s (1–550 aa N-term, 551–975 aa C-term) and full-length Mzt1 were cloned into pGAD424. t-Grip91s (1–966 aa N-term, 967–1932 aa C-term) were cloned into pGBT9 and the full-length γ-Tubulin23C was cloned into pGAD424 and pGBT9 vectors, using HiFi DNA Assembly Master Mix (E2621S, NEB) (Supplementary Figure 6A). N- and C-terminal parts of t-Grip84, t-Grip91 and t-Grip128 were inserted into the pDEST15 (11802014, Thermo Fisher) vector by Gateway cloning (11791-020, Thermo Fisher). To generate prey proteins for *in vitro* binding N- and C-terminal parts of t-Grip84, t-Grip91 and t-Grip128 were cloned into pJET1.2 plasmid downstream of the T7 promoter using CloneJET PCR Cloning Kit (K1231, Thermo Fisher). cDNA of Mzt1, γ-Tub23C were obtained from the Drosophila Gold collection (DGRC). Primers used in cloning are listed in Supplementary Figure 8.

### Staining and Microscopy

Dissection and staining of testis were performed as described by White-cooper (2004). Rat anti-HA (1:100) (11867423001, Roche), mouse anti-pan polyglycylated Tubulin (1:5,000) (clone AXO 49, MABS276, Merck), mouse anti-γ-Tubulin (1:5,000) (clone GTU-88, T6557, Sigma), rabbit anti-Ana1 (1:5,000) (Fu et al., 2016), rabbit anti-Asl (1:1,000) (Fu et al., 2016) primary antibodies were used. Secondary antibodies Alexa Fluor 488 (A-11029, A-21208) Alexa Fluor 546 (A-11030, A-11081, A-11010, A32754), and Alexa Fluor 647 (A-21052) conjugated anti-mouse, anti-rat, and anti-rabbit antibodies (Thermo Fisher), respectively, were used (1:400). Texas Red1-X Phalloidin (T7471, Life Technologies) was used at a 1:250 dilution. 4′,6-diamidino-2-phenylindole (DAPI) was used at 1 μg/ml concentration (D1306, Thermo Fisher). Mitotracker Red CMXRos (0.5 μM in PBS) (M7512, Thermo Fisher) and Hoechst (1:500 in PBS) (H3570, Thermo Fisher) were used on freshly dissected testes for a 20 min incubation period. Fixed samples were mounted in SlowFade Gold antifade reagent (S36967, Life Technologies). Images were taken using an Olympus BX51 fluorescent microscope or an Olympus Fluoview Fv10i Confocal microscope.

### Yeast Two-Hybrid Assay

Yeast two-hybrid assay was carried out using the Matchmaker two-hybrid system (K1605, Clontech). One of the baits pGBT9: N- and C- terminal parts of t-Grip91 and full-length γ-Tub23C was cotransformed with one of the preys pGAD424: N- and C- terminal parts of t-Grip84, t-Grip91, t-Grip128, full-length Mzt1 and γ-Tub23C into PJ69 yeast cells (James et al., 1996). Individual colonies were streaked out on yeast synthetic double drop-out plates that lacked tryptophan and leucine (Y0750, Merck). After growing, they were inoculated on quadruple dropout plates lack tryptophan, leucine, histidine, and adenine (Y2021, Merck) containing 10 mM 3-amino-1, 2, 4-aminotrizole (3-AT) (A8056, Sigma).

### Recombinant Protein Production

Recombinant GST-tagged bait proteins were expressed, purified, and immobilized onto glutathione sepharose beads as described previously (17-0756-01, GE Healthcare) (Karman et al., 2020).

### *In vitro* Transcription-Translation (IVTT) and *in vitro* Binding

^35^S-methionine-labelled prey proteins (N-terminal and C-terminal parts of t-Grip91, full-length Mzt1 and γ-Tub23C) were produced *in vitro* using the TnT T7 Quick Coupled IVTT kit (L1170, Promega). Detailed protocol of IVTT and the GST-binding assay was described previously (Karman et al., 2020).

### Quantitative RT-PCR

Total RNA was purified from 25 pairs of testes, for *t-Grip84^*ms*^*, *t-Grip91^*ms*^*, *t-Grip128^Δ65^* and WT using Quick-RNA MiniPrep kit (R1054, Zymo Research). For the first-strand cDNA synthesis, RevertAid First Strand cDNA Synthesis Kit (K1670, Thermo Fisher) was used according to the manufacturer’s instructions. Maxima SYBR Green/ROX qPCR Master Mix (K0222, Thermo Fisher) was used for the real-time quantitative PCR reaction, according to the manufacturer’s instructions. Reactions were run on three occasion times in triplicates in the Rotor-Gene Q Real-Time PCR Detection System (QIAGEN) with the following reaction conditions: 95°C 10 min, 50 cycles of 95°C 15 s, 54°C 30 s, 72°C 30 s. Gene-specific and *rp49* specific primers were used and are listed in Supplementary Figure 8.

### Alignments, Quantification and Statistical Analysis

Pairwise sequence alignments for paralogs were made by Clustal Omega 1.2.4. Alignments were visualised using Unipro UGENE 1.31.1. Domain predictions were highlighted based on Pfam predictions. The phylogenetic tree was constructed by MEGA X with the maximum likelihood method. Data analysis and graph production were carried out using Origin 9.0 (OriginLab Corporation, Northampton, MA, United States). One-way ANOVA followed by Tukey’s HSD test were used to produce comparisons between WT and mutants (Supplementary Figures 2A–C,G,H, 5D).

## Data Availability Statement

The raw data supporting the conclusions of this article will be made available by the authors, without undue reservation.

## Author Contributions

RS contributed to the conception and design of the study and wrote the first draft of the manuscript. EA, VV, ZR-N, ZL, and RS performed the experiments. EA and ZL wrote sections of the manuscript. All authors contributed to manuscript revision, read, and approved the submitted version.

## Conflict of Interest

The authors declare that the research was conducted in the absence of any commercial or financial relationships that could be construed as a potential conflict of interest.

## Publisher’s Note

All claims expressed in this article are solely those of the authors and do not necessarily represent those of their affiliated organizations, or those of the publisher, the editors and the reviewers. Any product that may be evaluated in this article, or claim that may be made by its manufacturer, is not guaranteed or endorsed by the publisher.
