# Supplementary material for: Microtubule Organizing Centers Contain Testis-Specific γ-TuRC Proteins in Spermatids of Drosophila
Source: Front Cell Dev Biol. 2021 Sep 29;9:727264. doi: 10.3389/fcell.2021.727264 (PMC8511327; doi:10.3389/fcell.2021.727264)
Supplement: Supplementary file 1 [file Image_1.pdf]

Supplementary Figure 1

Legend:

Breakpoints of N- and C-terminal fragments used in protein-protein interaction experiments

Predicted Pfam domains:

t-Grip128: 383-539 aa - Gamma tubulin complex component protein, N-terminal (GCP_N)
Grip128: 778-1076 aa - Gamma tubulin complex component, C-terminal (GCP_C)

t-Grip91: 1312-1590 aa - Gamma tubulin complex component protein, N-terminal (GCP_N)
Grip91: 235-544 aa - Gamma tubulin complex component protein, N-terminal (GCP_N)
549-880 aa - Gamma tubulin complex component, C-terminal (GCP_C)

t-Grip84: 96-397 aa - Gamma tubulin complex component protein, N-terminal (GCP_N)
402-710 aa - Gamma tubulin complex component, C-terminal (GCP_C)
Grip84: 202-495 aa - Gamma tubulin complex component protein, N-terminal (GCP_N)
498-838 aa - Gamma tubulin complex component, C-terminal (GCP_C)

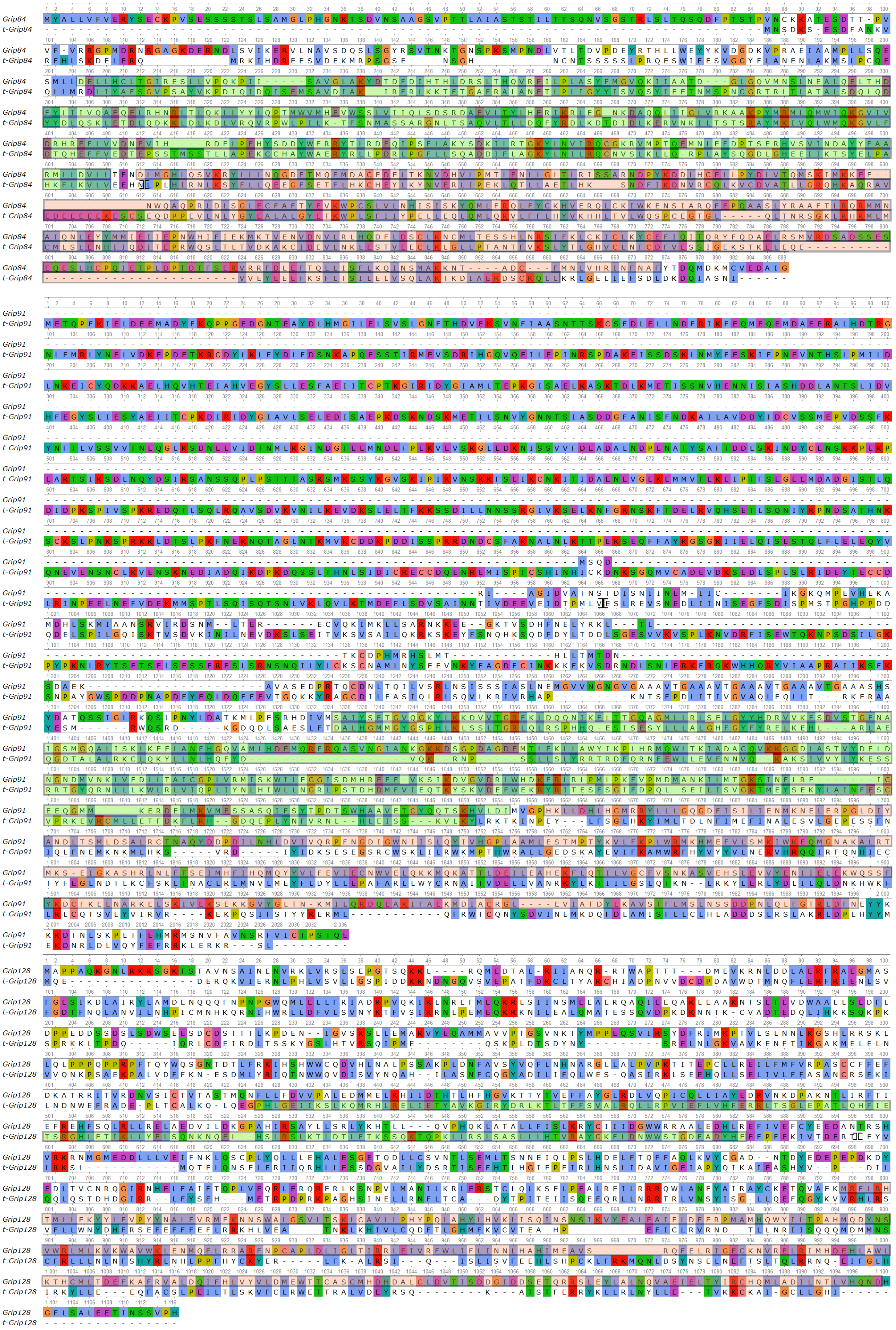

Supplementary Figure 1. Protein sequence alignment of of t-γ-TuRC members.

Pairwise protein sequence alignment of Grip84, t-Grip84, Grip91, t-Grip91 and Grip128, t-Grip128. Predicted Pfam domains are highlighted in green (Gamma tubulin complex component protein, N-terminal) and in red (Gamma tubulin complex component, C-terminal).
