# Supplementary material for: Microtubule Organizing Centers Contain Testis-Specific γ-TuRC Proteins in Spermatids of Drosophila
Source: Front Cell Dev Biol. 2021 Sep 29;9:727264. doi: 10.3389/fcell.2021.727264 (PMC8511327; doi:10.3389/fcell.2021.727264)
Supplement: Supplementary file 2 [file Image_2.pdf]

Supplementary Figure 2

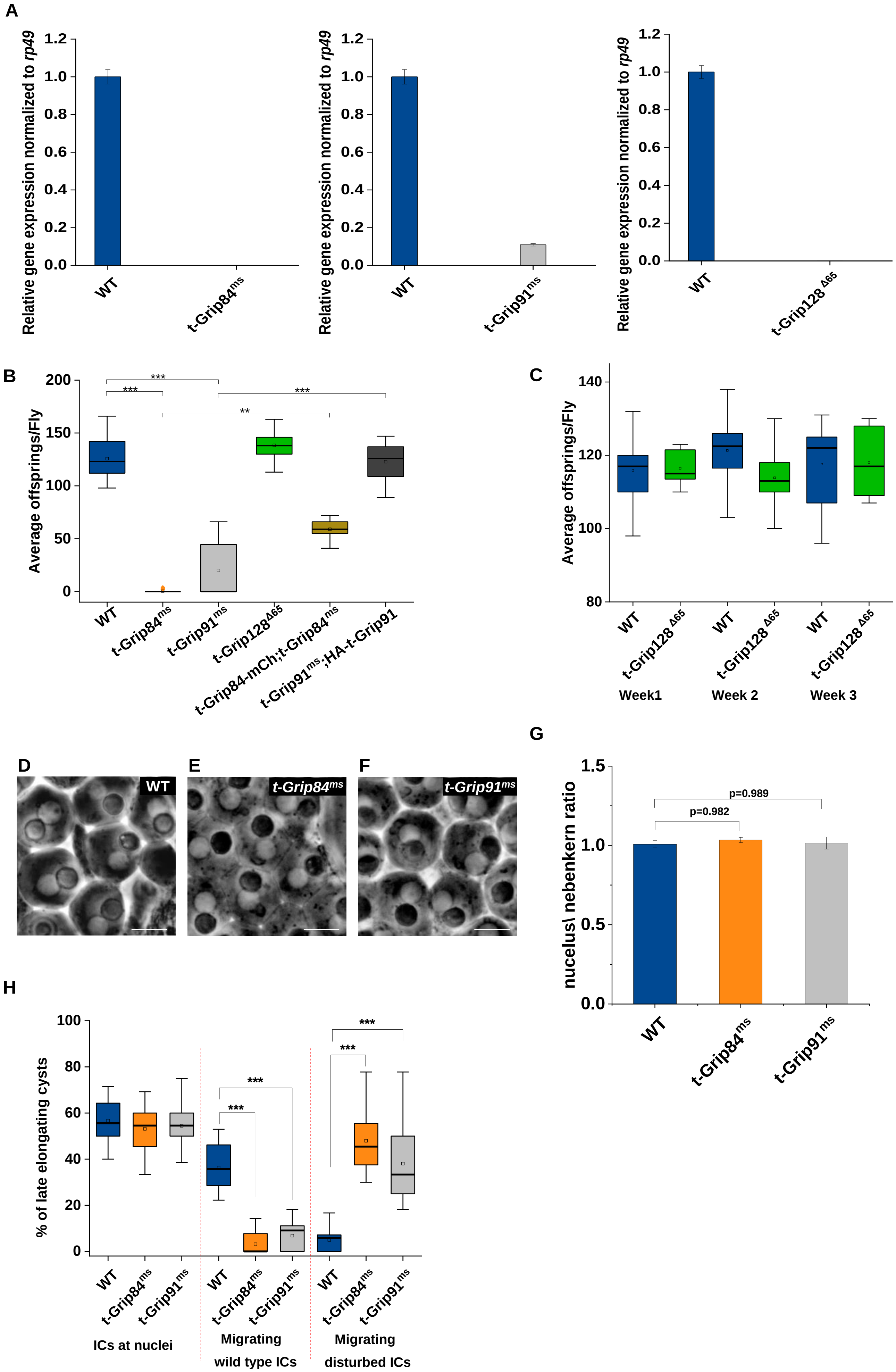

Supplementary Figure 2. Phenotypic characterization of t-γ-TuRC mutants.

(A) Relative mRNA expression was measured from dissected testes of WT, *t-Grip84^{ms}*, *t-Grip91^{ms}* and *t-Grip128$^{\Delta 65}$* males (n=25 pairs of testes). mRNA expression was normalized to *rp49*. **(B)** Box plots represent the fertility test of WT (fertile, n=27), *t-Grip84^{ms}* (sterile, n=27), *t-Grip91^{ms}* (semi-sterile, 70%, n=19) and *t-Grip128$^{\Delta 65}$* (fertile, n=20) mutants. t-Grip84-mCh transgene partially (47%) rescued the male-sterile phenotype of *t-Grip84^{ms}* males (n=21), while HA-t-Grip91 was able to rescue the male-sterile phenotype of *t-Grip91^{ms}* (n=12). $p<0.001$. Statistical significance was determined by one-way ANOVA. **(C)** t-*Grip128$^{\Delta 65}$* (n=20) fertility measured upon ageing of males for 3 weeks was normal compared to WT (n=20). **(D-F)** Phase-contrast images show onion-stage round spermatid cyst from WT, *t-Grip84^{ms}* and *t-Grip91^{ms}* mutant testes. **(G)** Graph shows the results of analysis of onion-stage round spermatid cyst from 1 day old adult of WT, *t-Grip84^{ms}* and *t-Grip91^{ms}* mutant. (n=50 cells in each genotype) **(H)** Individualization complex formation and movement were visualized with Phalloidin and DAPI staining in WT, *t-Grip84^{ms}* and *t-Grip91^{ms}* mutant testes (n=30 testes in each genotype) The number of ICs at the nuclei and normal or disturbed migration of ICs were counted in each genotype. Scale bars: D-F 10μm. Statistical significance was determined by one-way ANOVA ($p<0.001$).
