# Supplementary material for: Microtubule Organizing Centers Contain Testis-Specific γ-TuRC Proteins in Spermatids of Drosophila
Source: Front Cell Dev Biol. 2021 Sep 29;9:727264. doi: 10.3389/fcell.2021.727264 (PMC8511327; doi:10.3389/fcell.2021.727264)
Supplement: Supplementary file 3 [file Image_3.pdf]

Supplementary Figure 3

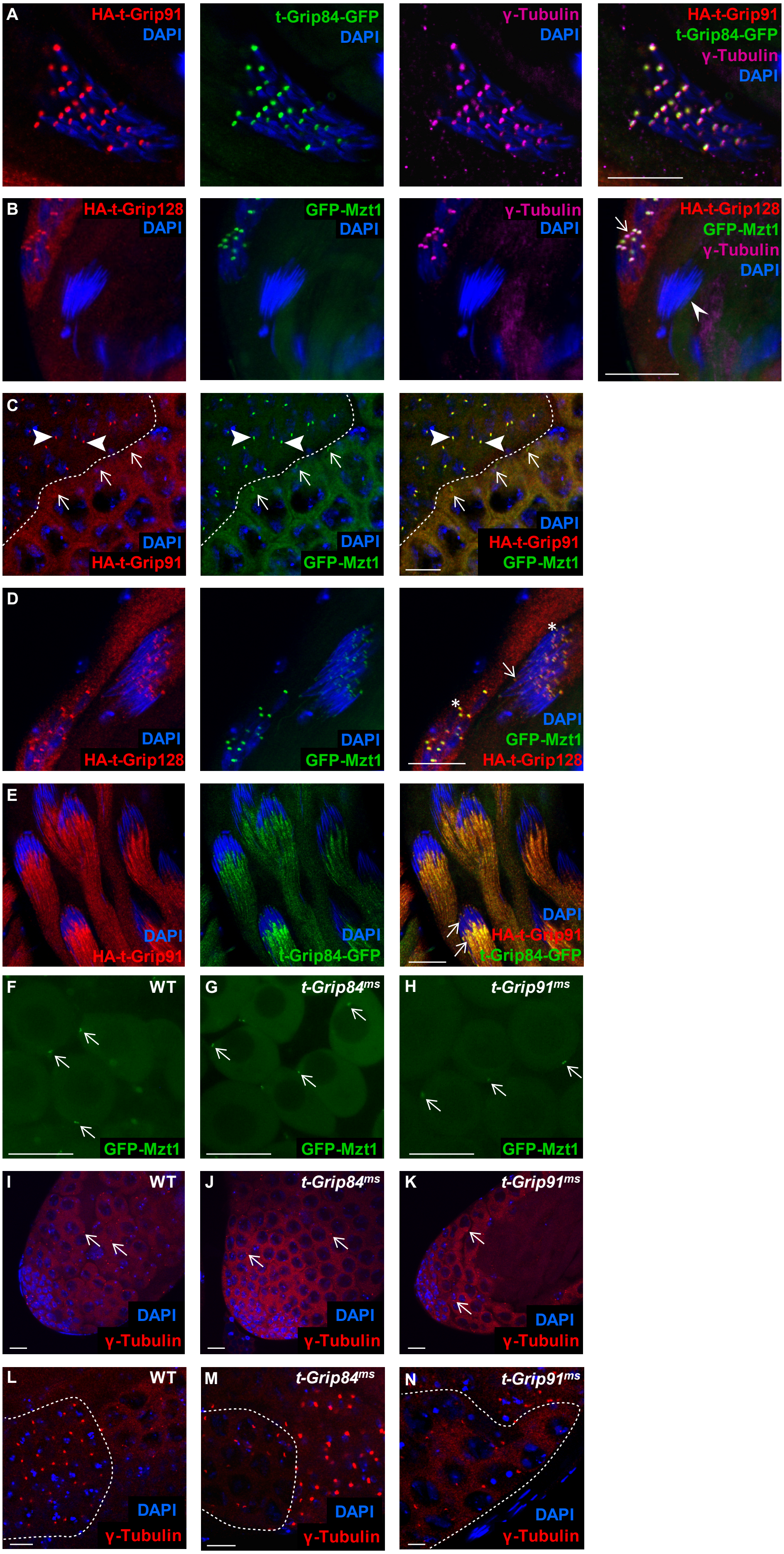

Supplementary Figure 3. Localization of t-γ-TuRC proteins and centriole adjunct components.

(A) HA-t-Grip91 and t-Grip84-GFP and γ-tubulin (magenta) are colocalizing on the centriole adjunct during nuclear elongation. **(B)** HA-t-Grip128 (red), GFP-Mzt1(green) and γ-tubulin (magenta) are colocalizing on the centriole adjunct of elongating spermatids (arrow), but not in the fully elongated ones (arrowhead). **(C)** HA-t-Grip91 localizes to the centriole adjunct in the post-meiotic spermatids (arrowhead) (the cyst highlighted with dashed line) and colocalize with GFP-Mzt1 to the centriole adjunct (arrowhead), but not to the meiotic centrosome (arrow). **(D)** HA-t-Grip128 colocalizing with GFP-Mzt1 on the centriole adjunct (asterisk) and on the tip of the nuclei of the elongating spermatids (arrow). **(E)** t-Grip84-GFP (green) colocalizes with HA-t-Grip91 (red) to the centriole adjunct and the tip of the nucleus (arrows) in late elongating spermatids. **(F-N)** GFP-Mzt1 (green) and γ-tubulin are localized to the centrosome (arrows) of spermatocytes in WT (F, I, L), *t-Grip84*^{ms} (G, J, M) and *t-Grip91*^{ms} (H, K, N) mutants. (individual cysts are highlighted by dashed lines) Scale bars: A -N 20μm.
