# Supplementary material for: Microtubule Organizing Centers Contain Testis-Specific γ-TuRC Proteins in Spermatids of Drosophila
Source: Front Cell Dev Biol. 2021 Sep 29;9:727264. doi: 10.3389/fcell.2021.727264 (PMC8511327; doi:10.3389/fcell.2021.727264)
Supplement: Supplementary file 4 [file Image_4.pdf]

Supplementary Figure 4

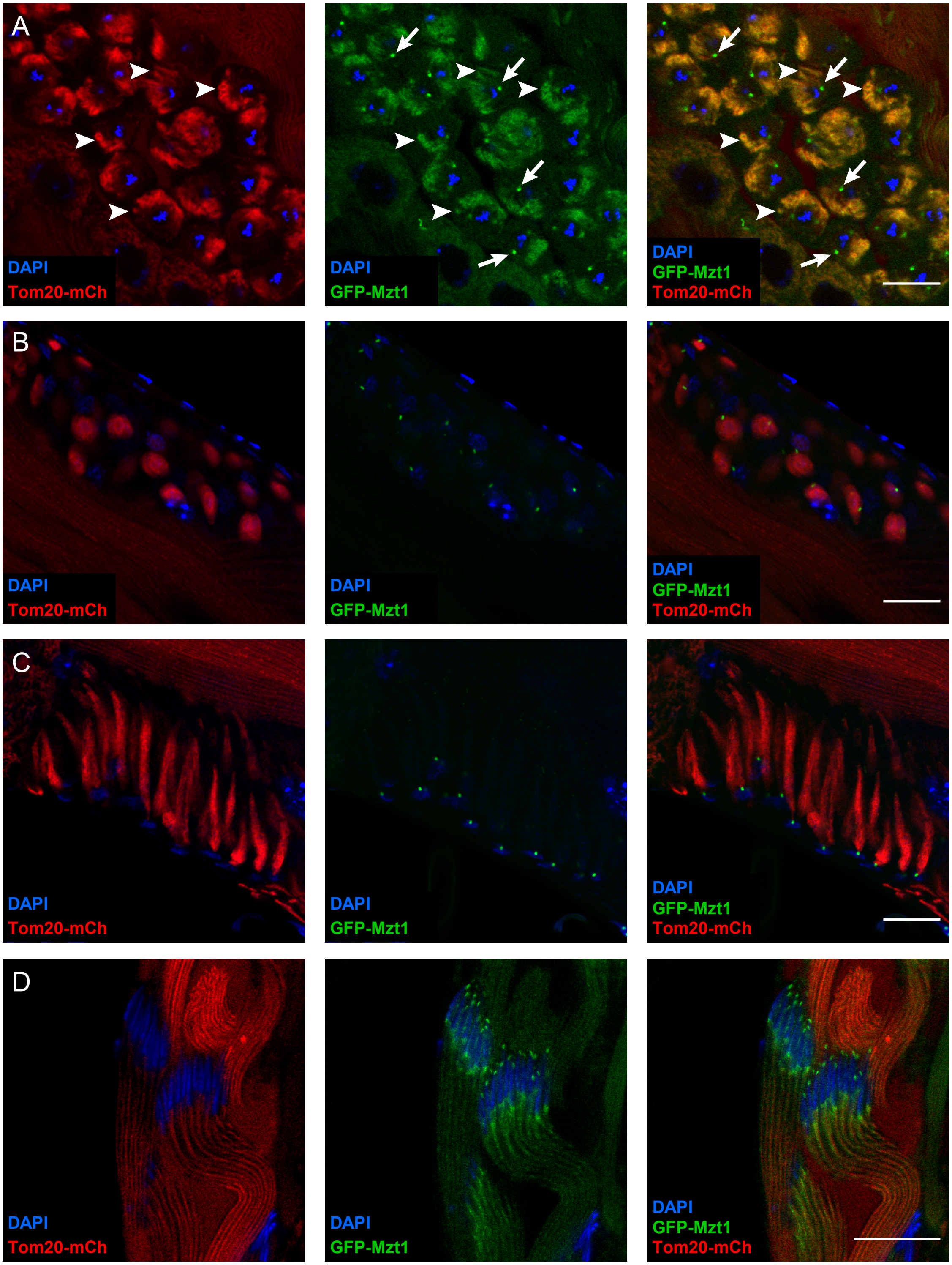

Supplementary Figure 4. Localization of GFP-Mzt1 in meiotic and post-meiotic stages of spermatogenesis

(A) GFP-Mzt1 (green) has an overlapping pattern with the mitochondrial marker Tom20-mCh (red, arrowheads) and also localize the centrosome (arrows) of meiotic spermatocytes **(B) (C)** In the round and early elongating spermatid stage GFP-Mzt1 accumulates on the basal body and centriole adjunct. **(D)** Late elongating spermatids accumulate GFP-Mzt1 at the apical tip of the nuclei and the centriole adjunct and in close proximity to the elongated mitochondria Scale bars: 20μm
