# Supplementary material for: Microtubule Organizing Centers Contain Testis-Specific γ-TuRC Proteins in Spermatids of Drosophila
Source: Front Cell Dev Biol. 2021 Sep 29;9:727264. doi: 10.3389/fcell.2021.727264 (PMC8511327; doi:10.3389/fcell.2021.727264)
Supplement: Supplementary file 5 [file Image_5.pdf]

Supplementary Figure 5

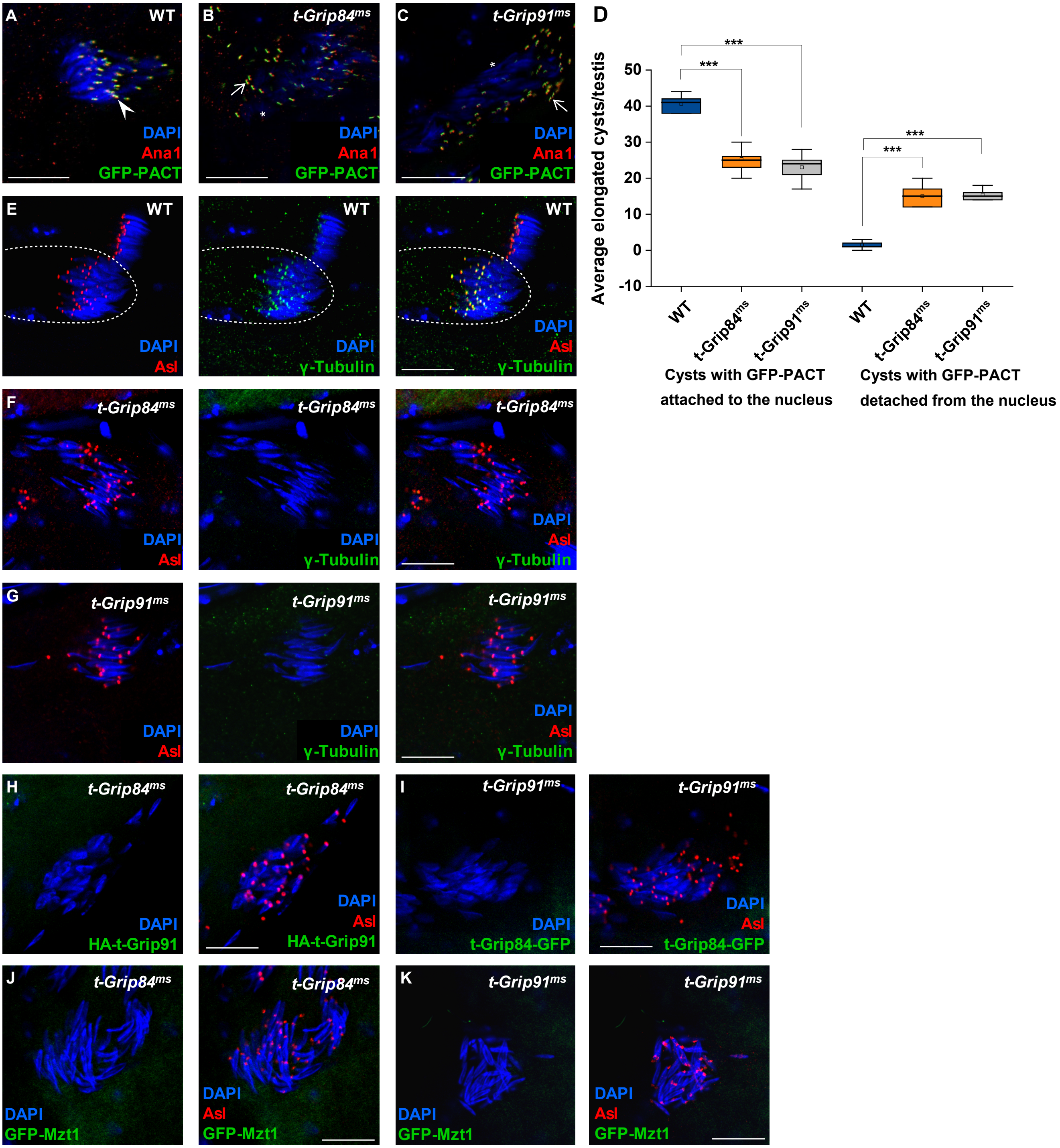

Supplementary Figure 5 (A-C) Ana1 (red) and GFP-PACT (green) are colocalizing in WT (A, arrowhead), *t-Grip84*^{ms} and *t-Grip91*^{ms} spermatids, but basal bodies are scattered in the elongated cysts of *t-Grip84*^{ms} and *t-Grip91*^{ms} mutants. (B, C arrows) **(D)** GFP-PACT signals and attachment to the nucleus were counted in the elongated cysts of WT and *t-Grip84*^{ms} and *t-Grip91*^{ms} mutants (n=12 pairs of testes in each genotype). **(E)** Asl and γ-Tubulin are colocalizing in WT spermatids **(F-K)** γ-Tubulin, HA-Grip91, GFP-Mzt lacking centriole adjunct has normal centriolar Asl localization is *t-Grip84*^{ms} (F, H, J) and *t-Grip91*^{ms} (G, I, K) mutants. (individual cysts are highlighted by dashed lines) Statistical significance was determined by one-way ANOVA ($p<0.001$).
Scale bars: 20μm
