# Supplementary material for: Microtubule Organizing Centers Contain Testis-Specific γ-TuRC Proteins in Spermatids of Drosophila
Source: Front Cell Dev Biol. 2021 Sep 29;9:727264. doi: 10.3389/fcell.2021.727264 (PMC8511327; doi:10.3389/fcell.2021.727264)
Supplement: Supplementary file 6 [file Image_6.pdf]

Supplementary Figure 6

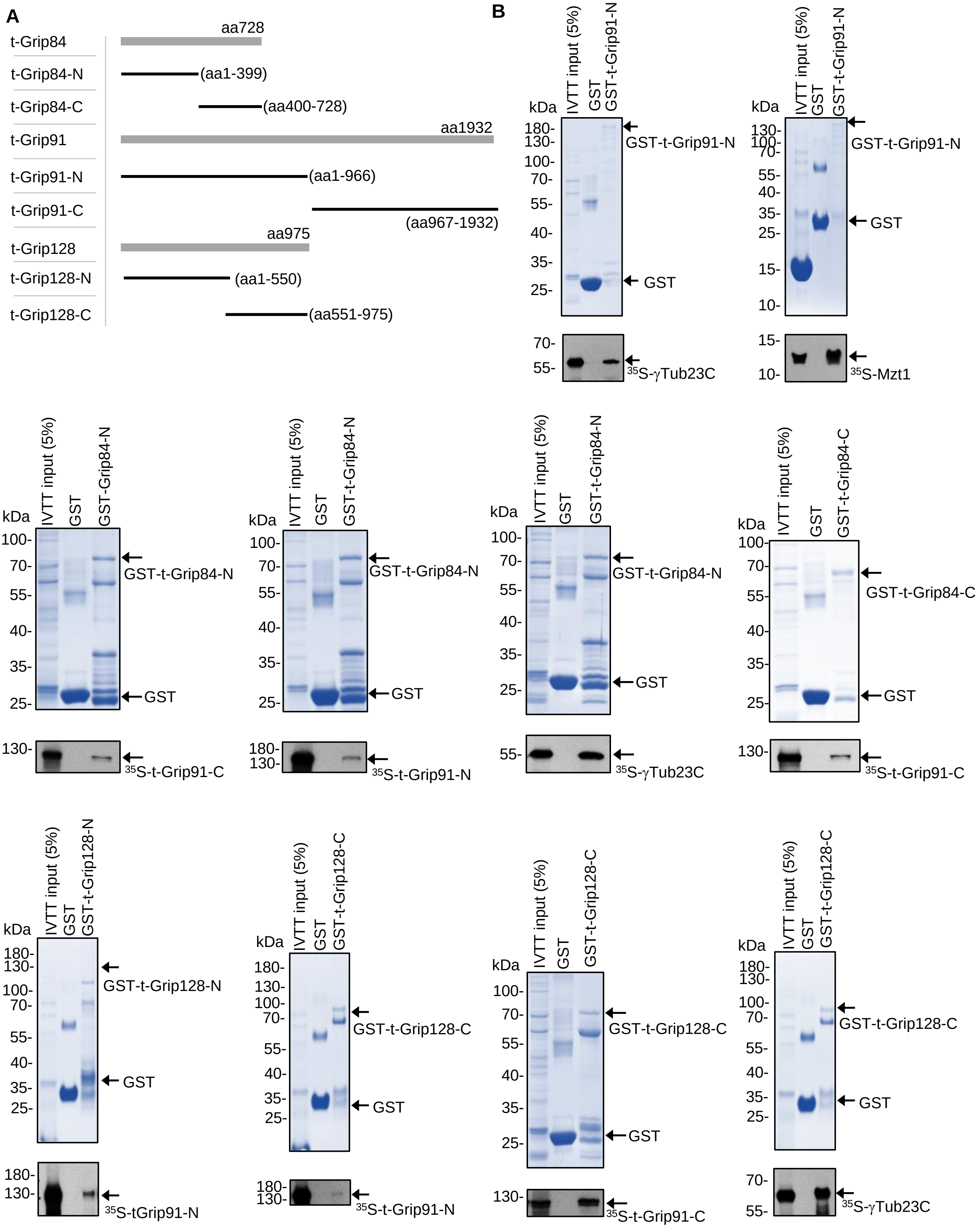

Supplementary Figure 6. t-γ-TuRC proteins fragments and IVTT interactions

(A) Schematic representation of t-γ-TuRC proteins the size of the N- and C-terminal fragments used in the Y2H assay and to express GST fusion proteins in bacteria. **(B)** Coomassie-Blue stained PAGE (upper panel) and autoradiographs (lower panel) of the ^{35}S labelled IVTT truncated (t-Grip91-N, t-Grip91-C) or full length of Mzt1 and γ-Tub23C proteins binding reaction with purified GST-t-Grip91-N, GST-t-Grip84-N, GST-t-Grip84-C, GST-t-Grip128-N and GST-t-Grip128-C.
