# Supplementary material for: Microtubule Organizing Centers Contain Testis-Specific γ-TuRC Proteins in Spermatids of Drosophila
Source: Front Cell Dev Biol. 2021 Sep 29;9:727264. doi: 10.3389/fcell.2021.727264 (PMC8511327; doi:10.3389/fcell.2021.727264)
Supplement: Supplementary file 7 [file Image_7.pdf]

Supplementary Figure 7

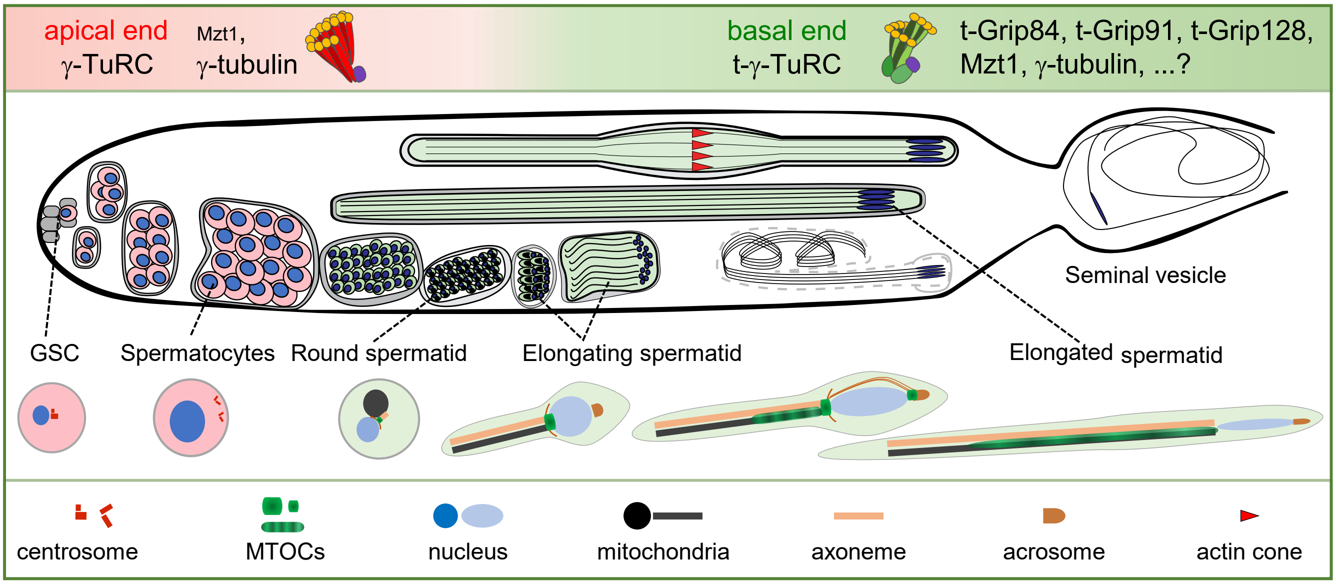

Supplementary Figure 7. γ-TuRC and MTOCs distribution in Drosophila testis

Summary of the various stages of spermatogenesis with highlighting the stage-specific MTOCs. Core γ-TuRC and its binding proteins localize to the centrosome in spermatocytes. From the round spermatid stage t-γ-TuRC, γ-Tubulin and Mzt1 accumulate first on the centriole adjunct and besides the centriole adjunct also on at the apical tip of the nuclei and on the surface of the elongating mitochondria of spermatids.
