# Supplementary material for: Microtubule Organizing Centers Contain Testis-Specific γ-TuRC Proteins in Spermatids of Drosophila
Source: Front Cell Dev Biol. 2021 Sep 29;9:727264. doi: 10.3389/fcell.2021.727264 (PMC8511327; doi:10.3389/fcell.2021.727264)
Supplement: Supplementary file 8 [file Image_8.pdf]

Supplementary Figure 8

**Oligonucleotides**
t-Grip84-mCh_fw: ACTGCGGAATTCTTGAGTGAGTGTGAGCAGTAG
t-Grip84-mCh_rev: ACTGCGGCTAGCATTGGAAGCTATTTGATCCTTATC
HA-t-Grip91_fw: TCCAGATTACGCTATGGAGACGCAGCCATTTAAAATTG
HA-t-Grip91_rev: ATGTCACACCACAGAAGTAAGGTTCATTAAGCTACCAAGGTGACCACG
HA-t-Grip128_fw: GTTTTCAATAAAAATGGATCTCCACCGCGGTGG
HA-t-Grip128_rev: TGTCCACACCACAGAAGTAAGGTTCTTAAATATGGCCGAGCAAACAGCC
GFP-Mzt1_fw: CTGTACAAGATGTCAGAACAACCGACACAAC
GFP-Mzt1_rev: CACAGAAGTAAGGTTCCTACAAAGTTGAGTCTGTTGATGC
t-Grip84-GFP_fw: GATATATTTTCTTATTCACCATGAACTCCGACAAATCGGAAAG
t-Grip84-GFP_rev: TTGCTCACCATTAGATATCTCGAGTGCGGC
YTH t-Grip91-N_fw: ATCGCCGGAATTCCCAATGGAGACGCAGCCATTTAAAATTG
YTH t-Grip91-N_rev: GGTCGACGGATCCCCTTAAACTAACATAGGGGTATCAATTTCAAC
YTH t-Grip91-C_fw: ATCGCCGGAATTCCCAATGGAAAGTCTTCGTGAAG
YTH t-Grip91-C_rev: GGTCGACGGATCCCCTTACAGTGATCTTTTCCTTTCC
YTH t-Grip84-N_fw: AAAGAGATCGAATTCCCAATGAACTCCGACAAATCG
YTH t-Grip84-N_rev: GGTCGACGGATCCCCTTAATTGTGCTCCTCCACC
YTH t-Grip84-C_fw: AAAGAGATCGAATTCCCAATGTTGCCTTTACACATTCG
YTH t-Grip84-C_rev: GGTCGACGGATCCCCTTAAATATTGGAAGCTATTTGATCC
YTH t-Grip128-N_fw: AAAGAGATCGAATTCCCAATGCAAGAGGACGAGAG
YTH t-Grip128-N_rev: GGTCGACGGATCCCCTTATCGTTCATCGGTCACAATC
YTH t-Grip128-C_fw: AAAGAGATCGAATTCCCAATGACAGAGTACGTCTTAAGAAAATC
YTH t-Grip128-C_rev: GGTCGACGGATCCCCTTAAATATGGCCGAGCAAAC
YTH γ-Tub23C_fw: AAAGAGATCGAATTCCCAATGCCAAGTGAAATAATTACTTTGCAG
YTH γ-Tub23C_rev: GGTCGACGGATCCCCCTAGGAACCGGCGCTGGTC
YTH Mzt1_fwd: AAAGAGATCGAATTCCCAATGTCAGAACAACCGACAC
YTH Mzt1_rev: GGTCGACGGATCCCCCTACAAAGTTGAGTCTGTTGATG
GST t-Grip91-N_fw: CGACGGTACCATGGAGACGCAGCCATTTAAAAT
GST t-Grip91-N_rev: GCGGCCGCTTAAACTAACATAGGGGTATCAATTTCAAC
GST t-Grip128-N_fw: CGACGGTACCATGCAAGAGGACGAGAGACA
GST t-Grip128-N_rev: GCGGCCGCTTATCGTTCATCGGTCACAATC
GST t-Grip128-C_fw: CGACGGTACCATGACAGAGTACGTCTTAAGAAAATC
GST t-Grip128-C_rev: GCGGCCGCTTAAATATGGCCGAGCAAACA
GST t-Grip84-N_fw: GAATTCAAATGAACTCCGACAAATCGGAAAGTG
GST t-Grip84-N_rev: GCGGCCGCTTAATTGTGCTCCTCCACC
GST t-Grip84-C_fw: GAATTCAAATGTTGCCTTTACACATTCG
GST t-Grip84-C_rev: GCGGCCGCTTAAATATTGGAAGCTATTTGATCC
t-Grip128 $^{\Delta 65}$ CRISPR1: TATATAGGAAAGATATCCGGGTGAACTTCGAAAACAAGTCTTAAACTCAGTTTTAGAGCTAGAAATAGCAAG
t-Grip128 $^{\Delta 65}$ CRISPR2: ATTTTAACTTGCTATTTCTAGCTCTAAAACGATTCCGTCTAAGTTGAGTCGACGTTAAATTGAAAATAGGTC

List of primers used in this study.
